# Retinal Vascular Caliber: 6 to 12 Months and 15 to 25 Years Following Hypertensive Pregnancy

**DOI:** 10.1161/HYPERTENSIONAHA.126.27171

**Published:** 2026-06-17

**Authors:** Hannah R. Cutler, Henner Hanssen, Christoph Hauser, Lukas Streese, Prenali D. Sattwika, Jamie Kitt, Annabelle McCourt, Katie Suriano, Yvonne Kenworthy, Annabelle Frost, Samuel Krasner, Adam J. Lewandowski, Winok Lapidaire, Paul Leeson

**Affiliations:** Division of Cardiovascular Medicine (H.R.C., P.D.S., J.K., A.M.C., K.S., Y.K., A.F., S.K., A.J.L., W.L., P.L.), University of Oxford, United Kingdom.; Nuffield Department of Primary Care Health Sciences (J.K.), University of Oxford, United Kingdom.; Nuffield Department of Women’s and Reproductive Health (A.F.), University of Oxford, United Kingdom.; Nuffield Department of Population Health (A.J.L.), University of Oxford, United Kingdom.; Department of Sport, Exercise and Health, University of Basel, Switzerland (H.H., C.H., L.S.).; Faculty of Health Care, Niederrhein University of Applied Sciences, Krefeld, Germany (L.S.).; Department of Internal Medicine, Faculty of Medicine, Public Health, and Nursing, Universitas Gadjah Mada, Indonesia (P.D.S.).; National Institute for Health Care and Research, Oxford Biomedical Research Centre, United Kingdom (P.L.).

**Keywords:** arterial pressure, blood pressure, body mass index, pregnancy, retinal vessel

## Abstract

**BACKGROUND:**

Women display retinal vessel caliber abnormalities after hypertensive pregnancy, but whether these predate or reflect vascular aging is unknown.

**METHODS:**

Retinal imaging was performed in 2 cohorts: 196 women at 6 to 12 months postpartum (167 hypertensive, 29 normotensive), and 105 women at 15 to 25 years postpartum (64 hypertensive, 41 normotensive), using identical imaging/analysis protocols (Imedos Health, GmbH). Central retinal arteriolar and venular equivalents were calculated, corrected for mean arterial pressure at the time of imaging, and compared across hypertensive and normotensive pregnancy groups using multivariable linear regression models adjusted for body mass index and time postpartum. *Z* tests were used to compare effect sizes across time points.

**RESULTS:**

At both time points, corrected central retinal arteriolar and venular equivalents were lower after hypertensive pregnancies compared with normotensive pregnancies (*P*<0.001). Between-group differences were larger at 6 to 12 months than 15 to 25 years postpartum for both arteriolar (β=−0.53 versus -0.15; *Z*=−4.19; *P*<0.001) and venular calibers (β=−0.50 versus β=−0.18, *Z*=−3.83; *P*<0.001), consistent with age-related reductions after normotensive pregnancy. Arteriovenous ratio was significantly different at 6 to 12 months postpartum (*P*<0.001), but not at 15 to 25 years postpartum. Differences were not explained by blood pressure at the time of imaging, but 6 to 12 months postpartum, related to early pregnancy blood pressure (β=0.01; *P*<0.001).

**CONCLUSIONS:**

Hypertensive pregnancy is associated with retinal vessel changes up to 25 years postpartum that predate age-related reductions and are associated with early pregnancy blood pressure.

**REGISTRATION:**

URL: https://www.clinicaltrials.gov; Unique identifier: NCT04273854 and NCT05434195.

**Novelty and Relevance**

**What Is New?**

This study is the first to analyze retinal imaging data from 2 independent cohorts of women at early and late postpartum stages using identical imaging protocols, allowing comparison of changes in the small arteries and veins of the retinal microcirculation at different life stages.

**What Is Relevant?**

A history of hypertensive pregnancy was associated with narrower retinal arteriolar and venular caliber at both postpartum time points compared with normotensive pregnancy. Differences between groups were attenuated at 15 to 25 years, likely reflecting age-related vascular changes in the normotensive group.

Notably, retinal vessel caliber at 6 to 12 months postpartum in women with hypertensive pregnancies was narrower than that observed in the normotensive group, even 15 to 25 years postpartum, and it was associated with early pregnancy blood pressure levels. These findings suggest that microvascular alterations are present early after hypertensive pregnancy and may reflect an accelerated vascular aging trajectory.

**Clinical/Pathophysiological Implications?**

Collectively, these results support the need for early surveillance and risk stratification in women with a history of hypertensive pregnancy. Further research is required to determine whether early targeted interventions may mitigate microvascular dysfunction and reduce the long-term risk of cardiovascular and multiorgan disease in this high-risk population.

Hypertensive pregnancy is associated with increased risks of chronic hypertension, heart failure, stroke, and cardiovascular disease later in life.^1^ Vascular dysfunction, characterized by structural and functional abnormalities of the macro- and microcirculation, is thought to underlie this risk, often preceding overt cardiovascular events.^2^

In some women, hypertensive pregnancy can exacerbate vascular dysfunction through placental-driven inflammatory and ischemic cascades, with effects that persist beyond pregnancy.^3^ However, it remains unclear whether this vascular dysfunction predates pregnancy, arises during pregnancy, or develops subsequently because of accelerated vascular aging and disease progression.^4^ Clarifying the timing and persistence of these changes after hypertensive pregnancy is important for understanding disease pathogenesis, improving risk stratification, and identifying windows for early intervention.

The retinal microvasculature can provide a unique, noninvasive window into systemic vascular health. Alterations in retinal vessel caliber have been associated with hypertension,^5^ cardiovascular risk,^6–8^ and hypertensive pregnancy,^9^ and recent evidence supports the potential of retinal microvascular biomarkers as clinically applicable predictors of future cardiovascular events.^6^

We, therefore, aimed to investigate the likely timing of the maximal impact of hypertensive pregnancy on the retinal vasculature. This involved comparing retinal vessel caliber during the first year postpartum after a hypertensive pregnancy to that seen after a normotensive pregnancy. We then studied how the relative difference between groups differs 2 to 3 decades later.

## Methods

### Data Availability

Data is available from the chief investigator, P.L., on reasonable request, subject to the approval of the Sponsor, University of Oxford, and the Trial Steering Committees.

### Study Design and Population

This pooled cohort study with cross-sectional comparisons of 2 independent samples was conducted between January 2020 and July 2024. Participants were recruited from 3 larger studies (for recruitment details, see Participant Flow Diagram in the Supplemental Material). Ethical and research governance approval were obtained for all studies (POP-HT [Physician Optimized Postpartum-Hypertension Treatment]: 19/LO/1901, CAREFOL-HT [Clinical Antenatal Randomised Study to Characterise Key Roles of Tetrahydrofolate in Hypertensive Pregnancies]: 21/WA/1069, HELPFUL [Hypertension Explored in Long-Term Postpartum Follow-Up of Later Life]: 22/LO/0781), and all participants provided informed consent.

#### 6- to 12- Month Cohort

The hypertensive pregnancy cohort at 6 to 12 months postpartum was drawn from both arms of the POP-HT trial,^10–13^ a single-center, 2-group parallel design, prospectively randomized open-blinded end point study comparing physician-optimized home blood pressure management and standard National Health Service (NHS) care immediately postpartum. Data collection for this study ended in November 2021, and all participants with retinal imaging data were included in the analysis. Detailed methodology for outcomes such as anthropometry and blood pressure has been described previously.^10–13^

The normotensive pregnancy cohort at 6 to 12 months postpartum was enrolled as a control group of the CAREFOL-HT, a trial testing the effects of tetrahydrofolate supplementation in pregnancy. Data collection for this study ended in January 2026, and the analysis included participants who completed a study visit before October 2024.

Demographic and pregnancy-related data, including age, ethnicity, prepregnancy body mass index, and medical history, were collected during pregnancy and postpartum. Antenatal information, including blood pressures were obtained from the standard 12-week booking appointment retrieved from medical records. Information on sex, race, and ethnicity was self-reported in line with the National Institute for Health Research categories.^14^

#### 15- to 25-Year Cohort

The hypertensive and normotensive cohorts at 15- to 25-years postpartum were enrolled in a study called HELPFUL. This study started recruiting in June 2023 and ended in June 2024. The participants in this study were originally recruited during the PVS (Preeclampsia Vascular Study) at 5 to 10 years postpartum.^15,16^

Demographic and pregnancy-related data, including age, ethnicity, prepregnancy body mass index, and medical history, were collected during pregnancy and postpartum. Antenatal information, including blood pressures were obtained from the standard 12-week booking appointment retrieved from medical records. Information on sex, race, and ethnicity was self-reported in line with the National Institute for Health Research categories.^14^

### Inclusion and Exclusion Criteria

Detailed criteria for the inclusion and exclusion of the hypertensive and normotensive cohorts are provided in the Supplemental Methods. Briefly, participants were excluded from both cohorts if they had preexisting hypertension before pregnancy or any significant disease or disorder that affected their ability to participate.

Women were included in the hypertensive cohort if they had a clinician-confirmed diagnosis of gestational hypertension or preeclampsia at delivery, according to the UK National Institute for Health and Care Excellence guidelines.^17^ Gestational hypertension was defined as blood pressure over 140/90 mm Hg on at least 2 occasions after 20 weeks of gestation, measured clinically. Preeclampsia required the same blood pressure criteria plus new-onset abnormalities, such as proteinuria or organ dysfunction.

Inclusion criteria for the normotensive cohort required documented blood pressure readings consistently below 140/90 mm Hg across all pregnancies, in addition to the presence of <2 moderate risk factors for hypertensive pregnancy, as defined by the National Institute for Health and Care Excellence guidelines.^17^ To minimize misclassification and to exclude anyone who might have had a hypertensive episode outside of the study window, a comprehensive assessment was undertaken, including detailed questionnaires, baseline clinical screening, and a review of medical records.

### Data Collection

All studies were completed at the John Radcliffe Hospital, Oxford University Hospitals NHS Foundation Trust, United Kingdom. All studies used standardized imaging protocols and data processing techniques. Data collection for all 3 studies was completed by the research team at the Cardiovascular Clinical Research Facility at the John Radcliffe Hospital, United Kingdom, and all retinal imaging data analysis was completed by the research team at the Department of Sport, Exercise, and Health at the University of Basel.

### Retinal Imaging Acquisition and Analysis

Retinal imaging was performed at both time points using the same imaging protocols. Images were captured using a calibrated Topcon TRC-NW8 fundus camera (Nikon D7000 sensor) by a team of trained investigators (J.K., A.M.C., H.R.C., P.D.S., and S.K.). Participants were rested in a dark room for a few minutes before images were taken to achieve natural pupil dilatation without pharmacological mydriasis. Optic-disc centered digital photographs of both fundi were captured at an angle of 45°, with the right eye chosen for analysis due to comparable retinal vessel characteristics between the right and left eyes.^18^

**Figure 1.**
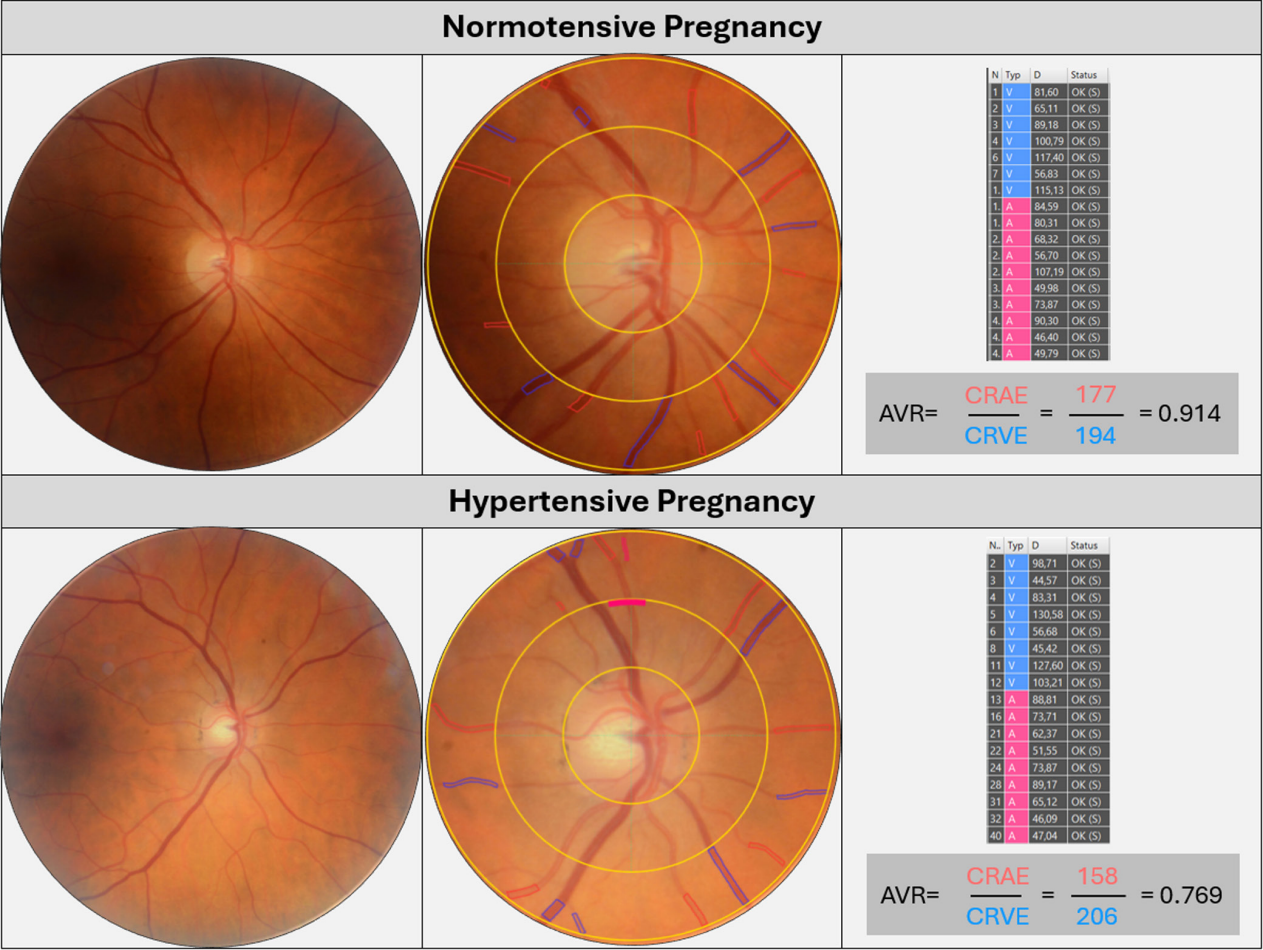
**Retinal vessel analysis in women with a history of either normotensive or hypertensive pregnancy.** Representative color fundus photographs and corresponding semi-automated retinal vessel analysis are shown for 1 woman with a history of normotensive pregnancy (**top** row) and 1 woman with a history of hypertensive pregnancy (**bottom** row). **Left**: original optic disc-centered fundus images. **Middle**: vessel segmentation and classification within standardized concentric measurement zones (yellow rings). Arteries are highlighted in red/pink and veins in blue. **Right**: vessel diameter measurements used to calculate central retinal artery equivalent (CRAE), central retinal vein equivalent (CRVE), and the arteriovenous ratio (AVR=CRAE/CRVE). The women with a hypertensive pregnancy history demonstrate narrower arterioles, reflected by a reduced CRAE and lower AVR compared with the normotensive women.

Retinal images were analyzed using semi-automated analysis software (Vesselmap 2; IMEDOS Health GmbH; Jena, Germany; Figure 1), following standard operating procedures.^19^ Briefly, vessels in the area of 0.5 to 1 disc diameter away from the optic disc margin were manually identified as arterioles or venules by an expert (L.S. and C.H.), automatically marked by the software, and manually reviewed and adjusted if necessary.

Retinal image analysis and quality checks were performed by experienced investigators (L.S., C.H., and H.H.) who were blinded to the clinical information of the participants. During the quality analysis checks, retinal images were excluded for reasons such as improper centering of the optic nerve head, incorrect image angles, or data extraction errors. The same retinal imaging analysts were used across all 3 studies, and the images were analyzed using identical protocols.

Arteriolar and venular diameters from 3 images per patient were used, where available, and averaged to central retinal arteriolar equivalent and central retinal venular equivalent using the Parr-Hubbard formula.^20^ The arteriolar-to-venular diameter ratio was calculated using the central retinal arteriolar equivalent and central retinal venular equivalent. Vessel diameters are presented in measuring units (mu). In the model of Gullstrand‘s normal eye, 1 mu relates to 1 µm. These standardized indices are widely used in population-based studies and facilitate comparison across cohorts.^6^

To account for the direct influence of intraluminal pressure on vessel diameter, retinal vessel caliber was corrected by dividing the central retinal arteriolar and venular equivalents by the mean arterial pressure, which was calculated using the following formula:


$$\begin{array}{cc} \; & Mean\;Arterial\;Pressure=Diastolic\;Pressure\\ \; & +\frac{1}{3}\;\left(Systolic\;Pressure-Diastolic\;Pressure\right). \end{array}$$

This is an approach that has been previously used to distinguish structural from pressure-dependent variation in vascular caliber.^21^ Arteriovenous ratio (arteriolar-to-venular diameter ratio) was then calculated by dividing the central retinal arteriolar equivalent by the central retinal venular equivalent. The arteriolar-to-venular diameter ratio represents the ratio of the central retinal arteriolar to venular diameter.

### Anthropometry and Blood Pressure Assessments

Height, weight, hip, waist, and mid left arm circumference were measured using standardized calibrated equipment, further details of which can be found in the Supplemental Methods. Clinical blood pressures were measured by a trained health care professional using an automated blood pressure machine (GE Dinamap Carescape V100, United Kingdom) with an appropriately sized cuff placed at least 2.5 cm above the elbow. Three measurements were taken 1 minute apart, and the average of the final 2 measurements was used, in accordance with current guidelines, across all studies.

### Statistical Analysis

Statistical analysis was conducted using R Foundation for Statistical Computing (version 4.4.2, Vienna, Austria) and R Studio (version 2024.12.0+467). The normality of the data and residuals was assessed using the Shapiro-Wilks test for continuous variables where n≤50 and by visual inspection of Q-Q plots and histograms for larger samples. Normally distributed continuous variables were reported as means±SD, while skewed continuous variables were reported as medians±interquartile range. Categorical data were presented as counts and percentages.

Baseline anthropometric and pregnancy characteristics were compared between normotensive and hypertensive groups using independent samples *t* tests for normally distributed continuous variables and Wilcoxon rank-sum tests for continuous variables with skewed distributions. Categorical variables were compared using Pearson χ^2^ tests or Fisher exact tests, as appropriate. Multicategory categorical variables, for example, ethnicity, were compared using a global Fisher exact test.

Multiple linear regression models, adjusted for body mass index and time postpartum, were used to compare retinal caliber characteristics across the hypertensive and normotensive groups at both time points. They were also used to examine any differences between women with preeclampsia and gestational hypertension.

*Z* tests were used to compare the effect sizes between the 6- to 12-month and 15 to 25 year postpartum cohorts. Ninety-five percent confidence intervals (CIs) were calculated for each effect size estimate.

To look for associations between antenatal blood pressures and retinal measurements, multiple linear regression models were used, adjusted for body mass index and time postpartum. Sensitivity analyses were conducted using 2-sample *t* tests to investigate the effect of physician-optimized home blood pressure monitoring versus standard NHS care postpartum on blood pressure and retinal outcomes within the hypertensive group.

## Results

### Study Cohort

In total, 301 participants were recruited for this analysis: 231 hypertensive and 70 normotensive women. Participant recruitment, follow-up, and inclusion in the final analytic sample are summarized in the participant flow diagram (Figure S1). Baseline demographic, anthropometric, pregnancy, and delivery characteristics at each time point are outlined and summarized in Table 1.

**Table 1.**
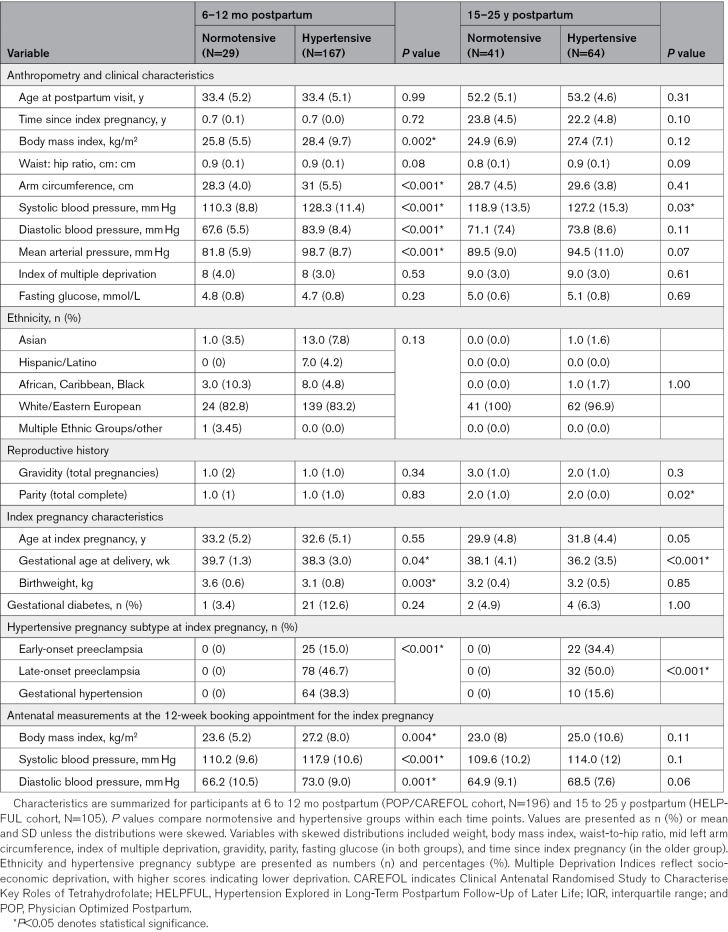
Anthropometric, Clinical, Demographic, Ethnic, Pregnancy, and Antenatal Characteristics of Participants at 6–12 Months and 15–25 Years Postpartum

#### 6- to 12-Month Postpartum

Of the 219 women who underwent retinal imaging at this time point, 23 data sets were excluded due to insufficient image quality (10.5%), leaving 196 women for analysis. This cohort included 167 women with previous hypertensive pregnancies and 29 with normotensive pregnancies. Within the hypertensive cohort, 103 women had a history of preeclampsia: 25 with early-onset disease (<34 weeks of gestation) and 78 with late-onset disease (>34 weeks of gestation). Meanwhile, 64 women had a history of gestational hypertension (no proteinuria or severe features).

Of the hypertensive women, 87 (52.1%) were randomized to receive physician- optimized home blood pressure management^10^ with 52 of these women having had preeclampsia and 35 having had gestational hypertension. The remaining 80 women (47.9%), including 51 with preeclampsia and 29 with gestational hypertension, received standard NHS care postpartum.

#### 15- to 25-Year Postpartum

120 women had retinal imaging at 15 to 25 years postpartum. Of these, 15 data sets were excluded due to image quality (12.5%), leaving 105 women, 64 of whom had hypertensive pregnancies and 41 had normotensive pregnancies. Of the hypertensive cohort, 54 (84.38%) were preeclamptic; 23 with early onset disease and 31 with late-onset disease, while 10 women (15.62%) had gestational hypertension during pregnancy.

### Retinal Caliber Characteristics at 6- to 12-Month Postpartum

The retinal caliber characteristics for both cohorts are summarized in Table 2. After adjustment for time postpartum and body mass index, corrected central retinal arteriolar equivalents were lower in women with hypertensive pregnancies compared with women with normotensive pregnancies (mean difference: 0.55 µm/mm Hg, adjusted *R*^2^=0.39, *P*<0.001).

**Table 2.**
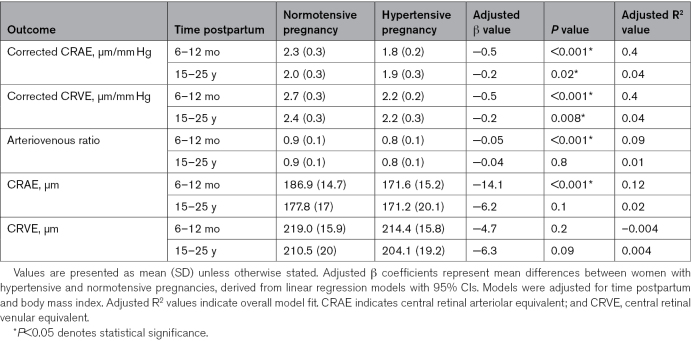
Retinal Caliber Characteristics at 6–12 Months and 15–25 Years Postpartum

Similarly, corrected central retinal venular equivalents were lower in women with hypertensive pregnancies compared with normotensive women (mean difference, 0.5 µm/mm Hg; adjusted *R*^2^=0.35, *P*<0.001) with a significant difference in arteriovenous ratio between groups (mean difference, 0.06; adjusted *R*^2^=0.09, *P*<0.001).

There were no differences in corrected central retinal venular equivalent (mean difference: 0.02 µm/mm Hg, adjusted *R*^2^=−0.01, *P*=0.88) between women with gestational hypertension and women with preeclampsia but the preeclamptic group showed a slightly higher corrected central retinal arteriolar equivalent compared with the gestational hypertension group (mean difference: 0.08 µm/mm Hg, adjusted *R*^2^=0.03, *P*=0.04), leading to a borderline lower arteriovenous ratio (mean difference: 0.02 µm/mm Hg, adjusted *R*^2^=0.03, *P*=0.06) in those previously diagnosed with preeclampsia compared with those previously diagnosed with gestational hypertension.

### Retinal Caliber Characteristics at 15- to 25-Year Postpartum

After adjustment for time postpartum and body mass index, corrected central retinal arteriolar equivalents were lower in the hypertensive group compared with the normotensive group at 15 to 25 years postpartum (mean difference: 0.15 µm/mm Hg, adjusted *R*^2^=0.04; *P*=0.02). Similarly, corrected central retinal venular equivalents were lower in the hypertensive group compared with the normotensive group (mean difference, 0.18 µm/mm Hg, adjusted *R*^2^=0.04; *P*=0.008). There were no differences in arteriovenous ratio between the hypertensive and normotensive cohorts (mean difference: 0.01 µm/mm Hg, adjusted *R*^2^=0.01; *P*=0.81). Due to the small number of women with gestational hypertension in this cohort, the differences between women with preeclampsia and gestational hypertension were not examined.

### Retinal Caliber Characteristics at 6 to 12 Months Compared With 15- to 25- Year Postpartum

**Figure 2.**
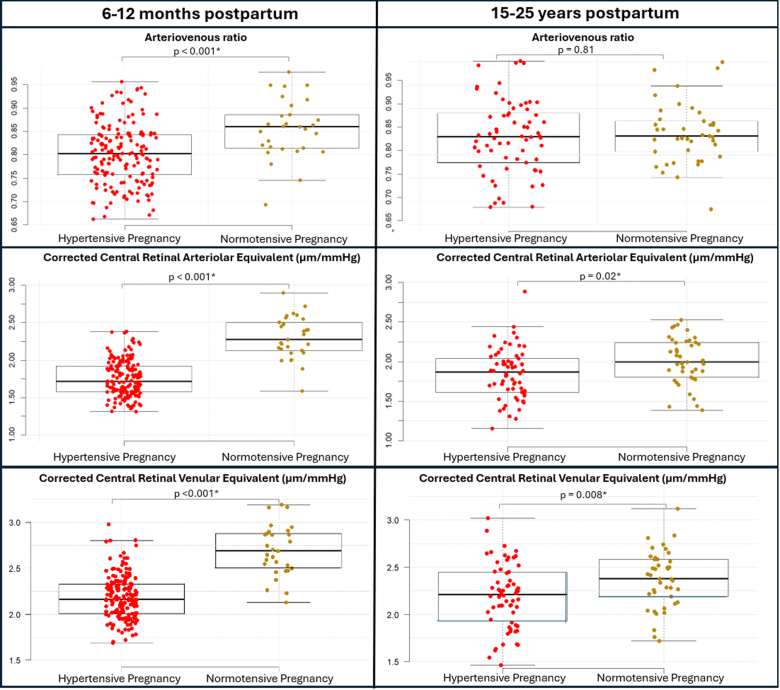
**Comparison of retinal caliber characteristics between hypertensive and normotensive groups at 2 postpartum time intervals.** The figure presents boxplots comparing the arteriovenous ratio, corrected central retinal arteriolar equivalent (CRAE), and corrected central retinal venular equivalent (CRVE) for hypertensive (red) and normotensive (gold) subjects at 6 to 12 months postpartum (**left**) and 15 to 25 years postpartum (**right**). Data are shown as boxplots with individual data points overlaid. *P<0.05 denotes statistical significance.

The absolute mean arteriolar sizes were comparable across time points for the hypertensive cohort (6–12 months: 172±15 µm/mm Hg; 15–25 years: 171±20 µm/mm Hg) but remained consistently lower than the normotensive group (Figure 2), despite there being differences in arteriolar size in the normotensive cohort across the 2 time points (6–12 months: 187±15 µm/mm Hg; 15–25 years: 178±17 µm/mm Hg).

The absolute mean venular sizes were smaller in both the normotensive and hypertensive pregnancy cohorts at the later postpartum time point but still lower in the hypertensive cohort at both time points (hypertensive 6–12 months: 214±16 µm/mm Hg, 15–25 years postpartum: 204±20 µm/mm Hg; normotensive 6–12 months: 219±16 µm/mm Hg, 15–25 years postpartum: 211±20 µm/mm Hg).

The difference in corrected central arteriolar equivalents between the hypertensive and normotensive cohorts was larger at 6 to 12 months postpartum (β=−0.53, SE=0.05 [95% CI, −0.63 to 0.43]) compared with 15 to 25 years postpartum (β=−0.15, SE=0.06 [95% CI, −0.27 to 0.03]), (*Z*=−4.19; *P*<0.001). The difference in corrected central venular equivalents was also larger at 6 to 12 months postpartum (β=−0.5, SE=0.05 [95% CI, −0.6 to 0.4]) than at 15 to 25 years postpartum (β=−0.18, SE=0.06 [95% CI, −0.3 to −0.06]; *Z*=−3.83; *P*<0.001). There was no significant difference in the effect sizes of the arteriovenous ratio between the 6 to 12 month cohort (β=−0.05, SE=0.01 [95% CI, −0.07 to −0.03]) and the 15 to 25 year cohort (β=−0.04, SE=0.01 [95% CI, −0.06 to −0.02]; *Z*=−0.61; *P*=0.54).

### Associations Between Retinal Caliber and Antenatal Blood Pressure

**Figure 3.**
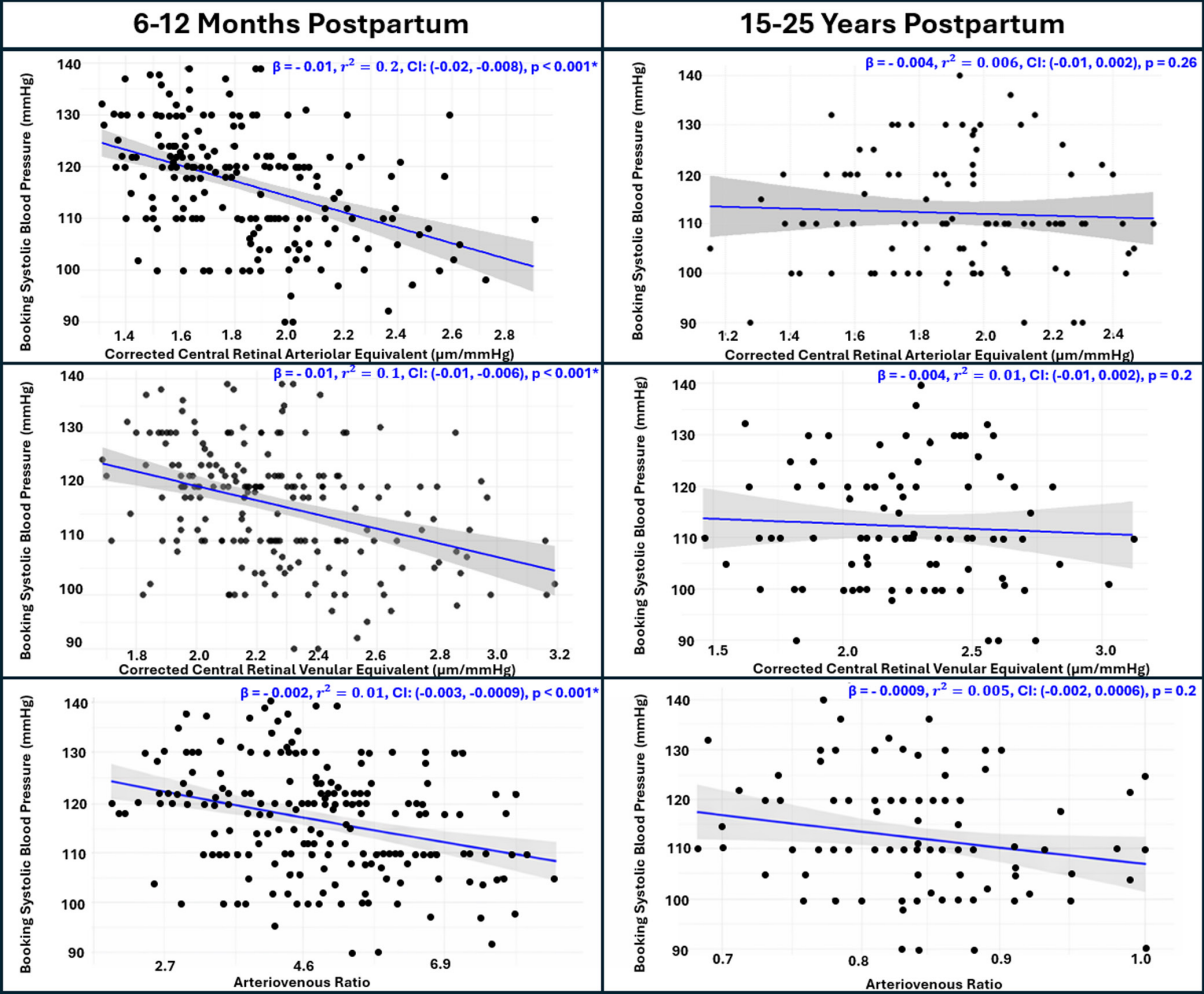
**Association between retinal microvascular measures at 6 to 12 months and 15 to 25 years postpartum and booking systolic blood pressure.** Scatter plots show the relationship between corrected central retinal arteriolar equivalent (CRAE), corrected central retinal venular equivalent (CRVE), and arteriovenous ratio (AVR) with booking systolic blood pressure (mm Hg), measured at the standard 12-week antenatal appointment. The **left** column represents measurements obtained at 6 to 12 months postpartum, and the **right** column represents measurements at 15 to 25 years postpartum. Each point represents an individual participant. Blue lines indicate linear regression fits, and shaded areas represent 95% CIs. Regression coefficients (β), coefficients of determination (r^2^), 95% CIs, and *P* values are displayed within each panel. *P<0.05 denotes statistical significance.

**Figure 4.**
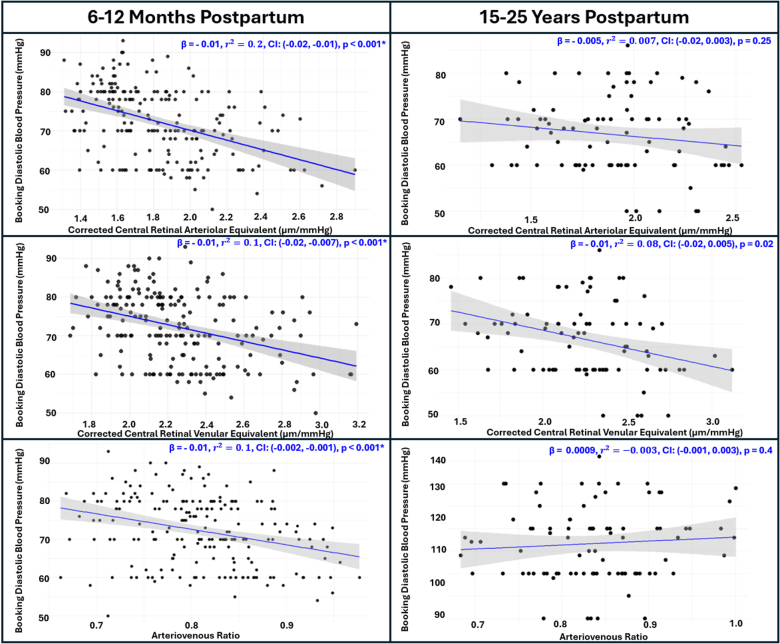
**Association between retinal microvascular measures at 6 to 12 months and 15 to 25 years postpartum and booking diastolic blood pressure.** Scatter plots show the relationship between corrected central retinal arteriolar equivalent (CRAE), corrected central retinal venular equivalent (CRVE), and arteriovenous ratio (AVR) with booking diastolic blood pressure (mm Hg), measured at the standard 12-week antenatal appointment. The **left** column represents measurements obtained at 6 to 12 months postpartum, and the **right** column represents measurements at 15 to 25 years postpartum. Each point represents an individual participant. Blue lines indicate linear regression fits, and shaded areas represent 95% CIs. Regression coefficients (β), coefficients of determination (r^2^), 95% CIs, and *P* values are displayed within each panel. *P<0.05 denotes statistical significance.

At 6 to 12 months postpartum, after adjustment for body mass index and time postpartum, corrected central retinal arteriolar equivalents were significantly inversely associated with antenatal systolic blood pressure (β=−0.02, adjusted *r*^2^=0.20 [CI, −0.02 to −0.008]; *P*<0.001) and diastolic blood pressure (β=−0.01, adjusted *r*^2^=0.21 CI, −0.02 to −0.01]; *P*<0.001), measured at the 12-week standard booking appointment (Figures 3 and 4).

Similarly, corrected central venular equivalents were significantly inversely associated with antenatal systolic blood pressure (β=−0.01, adjusted *r*^2^=0.13 [CI, −0.01 to −0.006]; *P*<0.001) and diastolic blood pressure (β=−0.01, adjusted *r*^2^=0.14 [CI, −0.02 to −0.007]; *P*<0.001). The arteriovenous ratio was also inversely associated with antenatal systolic blood pressure (β=−0.002, adjusted *r*^2^=0.01 [CI, −0.003 to −0.0009]; *P*<0.001) and diastolic blood pressure (β=−0.01, adjusted *r*^2^=0.1 [CI, −0.002 to −0.001]; *P*<0.001).

At 15 to 25 years postpartum, after adjustment for body mass index and time postpartum, there were no associations between corrected central retinal arteriolar equivalents and antenatal systolic blood pressure (β=−0.004, adjusted *r*^2^=0.006 [CI, −0.01 to −0.003]; *P*=0.26), nor diastolic blood pressure (β=−0.005, adjusted *r*^2^=0.007 [CI, −0.01 to −0.003; *P*=0.25).

There were also no associations between corrected central venular equivalents and antenatal systolic blood pressure (β=−0.004, adjusted *r*^2^=0.01 [CI, −0.01 to 0.002]; *P*=0.2). However, there was an inverse association with diastolic blood pressure (β=−0.01, adjusted *r*^2^=0.08 [CI, −0.02 to −0.005]; *P*=0.02). Additionally, there were no associations between the arteriovenous and antenatal systolic blood pressure (β=−0.0009, adjusted *r*^2^=0.005 [CI, −0.002 to 0.0006]; *P*=0.22), nor diastolic blood pressure (β=0.0009, adjusted *r*^2^=−0.0033 [CI, −0.001 to 0.003]; *P*=0.351).

### Subgroup Analysis of Physician Optimized Blood Pressure Management Intervention

Within the subgroup of participants in the POP-HT study there were no differences in corrected central arteriolar equivalents (mean difference: 0.04, *t*=1.06; *P*=0.29), corrected central venular equivalents (mean difference: 0.02, *t*=0.68; *P*=0.5) and arteriovenous ratios (mean difference: 0.09, *t*=0.91; *P*=0.36) between those who received physician-optimized home blood pressure management and those who received standard NHS care postpartum.

## Discussion

This study examined the associations between a history of hypertensive pregnancy and retinal vascular caliber at 2 postpartum time points. Retinal arteriolar and venular caliber were narrower in women with a prior hypertensive pregnancy at both 6 to 12 months and 15 to 25 years postpartum, independent of blood pressure, body mass index, and time since index pregnancy. The difference was greatest at 6 to 12 months postpartum, where the caliber reduction in the hypertensive pregnancy group exceeded that observed in the normotensive group 15 to 25 years postpartum. By 15 to 25 years postpartum, between-group differences were smaller but still evident. Additionally, retinal caliber at 6 to 12 months postpartum was associated with blood pressures measured in early pregnancy.

When comparing time points, the magnitude of the early postpartum differences is notable (Table 2). At 6 to 12 months postpartum, when the mean age was ≈30 years, women with a prior hypertensive pregnancy had a mean central retinal arteriolar equivalent of 172 µm, which is lower than the reported normative values for women over 55 years of age,^22^ and lower than the normotensive group at 15 to 25 years postpartum in this study (Table 2). Although cross-study comparisons should be interpreted cautiously, this pattern suggests that retinal arteriolar narrowing is evident soon after pregnancy and exceeds that expected from chronological aging alone.

The attenuation of relative effect sizes between groups at 15 to 25 years postpartum and limited power to detect differences in the arteriovenous ratio (Figure 2), likely reflects vascular aging in the normotensive group. Age-related reductions in retinal caliber, particularly in the venules, are well described in the literature and may relate to venous stiffening, reduced elasticity, and hormonal influences.^23,24^ In effect, vascular aging in women with prior normotensive pregnancy appeared to catch up to the underlying level observed in those with a hypertensive pregnancy. Nevertheless, retinal vascular differences remained detectable at 15 to 25 years postpartum, with women who had a hypertensive pregnancy still displaying relatively smaller retinal vascular calibers (Figure 2). These findings suggest a persistent microvascular phenotype, which would compound later risk.

Several, not mutually exclusive, mechanisms may underlie the observed narrowing.^25^ These include increased basal vascular tone due to endothelial dysfunction, heightened vasoconstrictor activity, or reduced nitric oxide bioavailability, as well as inward structural remodeling of the microvasculature, as seen in chronic hypertension.^25^ Such remodeling may persist beyond pregnancy and contribute to long-term disease risk. It is plausible that both functional and structural mechanisms coexist. However, as vessel caliber was assessed only under resting conditions, we were unable to differentiate between these processes. Studies incorporating maximal vasodilation testing are needed to clarify the underlying mechanisms.

The observation of narrower retinal arteriolar and venular caliber in women with prior hypertensive pregnancies (Table 2) may also have broader cardiovascular implications.^6^ Although derived from larger branch vessels, retinal vascular caliber is widely interpreted as a noninvasive, accessible marker of systemic microvascular health and likely reflective of broader microvascular alterations.^26^ Systemic microvascular dysfunction, including endothelial impairment, increased vasoconstrictor tone, and microvascular rarefaction, can increase peripheral vascular resistance, raise afterload, and promote left ventricular hypertrophy through chronic pressure overload.^27^ Over time, such alterations have been implicated in the pathogenesis of cardiovascular conditions such as heart failure with preserved ejection fraction.^28^ Although we did not directly assess microvascular rarefaction, endothelial function, or myocardial structure, mechanistic inferences should be made cautiously.

Furthermore, retinal caliber at 6 to 12 months postpartum was significantly associated with blood pressures measured at the 12-week antenatal booking visit (Figures 3 and 4). Although causality cannot be established, this finding raises the possibility that vascular differences precede pregnancy or reflect shared biological pathways contributing both to early blood pressure elevation and to the development of hypertensive pregnancy. Alternatively, factors related to abnormal placentation may reflect preexisting differences in vascular function. These hypotheses require prospective mechanistic investigation.

Finally, no differences in retinal vascular caliber were observed between women who did and did not receive the postpartum blood pressure intervention. However, the intervention was delivered over a relatively short postpartum period during a particular time of biological remodeling. As such, these findings do not allow firm conclusions regarding the effect of blood pressure control over longer time periods. Further studies with longitudinal blood pressure data and adequately powered designs are needed to clarify these relationships.

### Limitations

Several limitations should be considered when interpreting these findings. First, the observational design precludes causal inference, and residual confounding may persist despite adjustment for key variables, such as blood pressure, body mass index, and time postpartum.

Second, the incomplete retinal imaging data and relatively small normotensive cohort at 6-12 months postpartum may have reduced statistical power. The single-center design and relatively homogeneous study population also limited ethnic diversity and constrained generalisability. Larger, multi-center cohorts with more ethnically and socioeconomically diverse populations would confirm whether our findings are consistent across broader groups.

Third, although assessments were conducted at 2 postpartum time points, the study was not longitudinal in design. This prevented the evaluation of within-individual trajectories and the temporal sequence of retinal vascular changes. Consequently, we cannot determine whether the observed differences represent preexisting vascular phenotypes, progressive remodeling, or both. Prospective longitudinal studies incorporating serial retinal imaging and vascular function assessments will be necessary to clarify causality and life-course patterns.

Fourth, retinal vascular caliber reflects structural features and does not directly measure microvascular function. Retinal blood flow, neurovascular coupling, endothelial function, or systemic microvascular rarefaction were not assessed. Therefore, the functional and physiological implications of the observed morphological differences remain uncertain. Multimodal retinal and systemic vascular assessments will be necessary to establish whether these structural differences translate into clinically meaningful alterations in vascular function.

Fifth, the data were derived from multiple studies, including an intervention trial, which may introduce some heterogeneity. Nonetheless, all studies adhered to the same imaging and analysis protocols, which were performed by the same researchers to ensure consistency across the data sets.

Finally, participation at 15 to 25 years postpartum may reflect a healthier or health-conscious subset who are willing to reengage decades after pregnancy. This could have introduced participation bias and potentially attenuated observed differences at later time points. Also, it is possible that, in the younger cohort, some normotensive participants may develop hypertensive pregnancies in the future, and misclassification cannot be entirely excluded. However, this risk is low, as hypertensive pregnancy disorders most commonly occur in first pregnancies.

### Conclusions

Women with a history of hypertensive pregnancy show increased retinal vascular narrowing at 6-12 months and 15 to 25 years postpartum, compared with women with normotensive pregnancies. The effect size across groups is smaller at the later time point but at 6 to 12 months, the reduction in retinal caliber in those with a hypertensive pregnancy is already greater than that seen in women 15 to 25 years older who had a normotensive pregnancy. These findings indicate that vascular impairments extend beyond the immediate postpartum period and may reflect underlying vascular susceptibility in this population. In view of the association with early pregnancy blood pressure, it is possible that retinal vascular differences are already present in early gestation, before the clinical diagnosis of hypertensive disorders. However, this would need further investigation. Collectively, these observations highlight the importance of early identification and risk stratification to prevent later disease. Nonetheless, future research with longitudinal cohorts is needed to clarify the trajectory of these vascular changes.

### Perspectives

These findings suggest that changes in vascular caliber may reflect persistent, rather than transient, dysfunction, underscoring the importance of early prevention after hypertensive disorders of pregnancy. Further longitudinal studies are needed to better define the trajectory of these changes.

## Article Information

### Author Contributions

P. Leeson, AJ. Lewandowski, W. Lapidaire, K. Suriano, J. Kitt, A. Frost, S. Krasner, H.R. Cutler, and Y. Kenworthy contributed to the design of POP-HT (Physician Optimized Postpartum-Hypertension Treatment), CAREFOL-HT (Clinical Antenatal Randomised Study to Characterise Key Roles of Tetrahydrofolate in Hypertensive Pregnancies), or HELPFUL (Hypertension Explored in Long-Term Postpartum Follow-Up of Later Life). P. Leeson, W. Lapidaire, K. Suriano, Y. Kenworthy, H. Hanssen, C. Hauser, L. Streese, and A.J. Lewandowski refined the overall study protocol, led project delivery, and provided guidance and external refinement. Data collection and analysis were performed by J. Kitt, A. McCourt, H.R. Cutler, P.D. Sattwika, Y. Kenworthy, and S. Krasner. Retinal imaging analysis and quality checks were performed by H. Hanssen, C. Hauser, and L. Streese. H.R. Cutler performed the statistical analysis. H.R. Cutler and C. Hauser produced the tables and figures for the main article and Supplemental Material. H.R. Cutler and P. Leeson wrote the article. All authors contributed, revised, read, and accepted the final article.

### Disclosures

P. Leeson is a founder and shareholder in a medical device company and is a named inventor on granted patents related to cardiovascular imaging. P. Leeson has received consulting, advisory board, and speaker fees from Ultromics, AstraZeneca, Bracco, Daiichi Sankyo, and Pfizer. P. Leeson has been an investigator on research funds received from Merck, Osler Diagnostics, Ultromics, Novartis, Cytokinetics, and NovoNordisk. The other authors report no conflicts.

### Supplemental Material

Supplemental Methods

Figure S1

## Supplementary Material



## References

[1] Cutler HR Barr L Sattwika PD Frost A Alkhodari M Kitt J Lapidaire W Lewandowski AJ Leeson P. ‘Temporal patterns of pre- and post-natal target organ damage associated with hypertensive pregnancy: a systematic review’. Eur J Prev Cardiol. 2024;31:77–99. doi: 10.1093/eurjpc/zwad275 37607255 10.1093/eurjpc/zwad275 PMC10767256

[2] Liberale L Duncker DJ Hausenloy DJ Kraler S Bøtker H Podesser B Heusch G Kleinbongard P,. “Vascular (dys)function in the failing heart,”. Nat Rev Cardiol. 2025;22:728–750. doi: 10.1038/s41569-025-01163-w 40544172 10.1038/s41569-025-01163-w

[3] Palatnik A Kulinski J. ‘Hypertensive disorders of pregnancy & vascular dysfunction’. Front Cardiovasc Med. 2024;11:1411424. doi: 10.3389/fcvm.2024.1411424 38883989 10.3389/fcvm.2024.1411424 PMC11177763

[4] Enkhmaa D Wall D Mehta PK Stuart JJ Rich-Edwards JW Merz CNB Shufelt C. Preeclampsia and vascular function: a window to future cardiovascular disease risk. J Women’s Health. 2016;25:284–291. doi: 10.1089/jwh.2015.5414 10.1089/jwh.2015.5414 PMC4790201 26779584

[5] Klein R Klein BE Moss SE. ‘The relation of systemic hypertension to changes in the retinal vasculature: the Beaver Dam Eye Study’. Trans Am Ophthalmol Soc. 1997;95:329–48; discussion 348. 9440178 PMC1298366

[6] Hanssen H Streese L Vilser W. ‘Retinal vessel diameters and function in cardiovascular risk and disease.’. Prog Retin Eye Res. 2022;91:101095. doi: 10.1016/j.preteyeres.2022.101095 35760749 10.1016/j.preteyeres.2022.101095

[7] Wong TY Klein R Sharrett AR Duncan B Couper D Tielsch J Klein B Hubbard L. ‘Retinal arteriolar narrowing and risk of coronary heart disease in men and women: the Atherosclerosis Risk in Communities Study’. J Am Med Assoc. 2002;287:1153–1159. doi: 10.1001/jama.287.9.1153 10.1001/jama.287.9.1153 11879113

[8] Seidelmann SB Claggett B Bravo PE Gupta A Hoshang F Klein B Klein R Di Carli M Soloman S. ‘Retinal vessel calibres in predicting long-term cardiovascular outcomes: the Atherosclerosis Risk in Communities Study’. Circulation. 2016;134:1328–1338. doi: 10.1161/CIRCULATIONAHA.116.023425 27682886 10.1161/CIRCULATIONAHA.116.023425 PMC5219936

[9] Benschop L Schalekamp-Timmermans S Roeters van Lennep JE Jaddoe VWV Wong TY Cheung CY Steegers EAP Ikram MK. Gestational hypertensive disorders and retinal microvasculature: the Generation R Study. BMC Med. 2017;15:153. doi: 10.1186/s12916-017-0917-2 28803548 10.1186/s12916-017-0917-2 PMC5554975

[10] Kitt J Frost A Mollison J Tucker K Suriano K Kenworthy Y McCourt A Woodward W Tan C Lapidaire W. ‘Post-partum blood pressure self-management following hypertensive pregnancy: protocol of the POP-HT trial’. Br Med J Open. 2022;12:e051180. doi: 10.1136/bmjopen-2021-051180 10.1136/bmjopen-2021-051180 PMC8867381 35197335

[11] Kitt J Fox R Frost A Shanyinde M Tucker K Bateman P Suriano K Kenworthy Y McCourt A Woodward W. ‘Long-term blood pressure control after hypertensive pregnancy following physician-optimized self-management: the POP-HT randomized clinical trial’. J Am Med Assoc. 2023;330:1991–1999. doi: 10.1001/jama.2023.21523 10.1001/jama.2023.21523 PMC10640702 37950919

[12] Kitt J Krasner S Barr L Frost A Tucker K Bateman PA Suriano K Kenworthy Y Lapidaire W Lacharie M. ‘Cardiac remodeling after hypertensive pregnancy following physician-optimized blood pressure self-management: the POP-HT randomized clinical trial imaging substudy’. Circulation. 2024;149:529–541. doi: 10.1161/CIRCULATIONAHA.123.067597 37950907 10.1161/CIRCULATIONAHA.123.067597

[13] Kitt J Biasiolli L Krasner S Bateman PA Cutler HR Barr LC Frost A Tucker KL Suriano K Kenworthy Y. ‘Impact of blood pressure self-management on vascular remodeling after hypertensive pregnancy’. Hypertension. 2025;82:1938–1947. doi: 10.1161/HYPERTENSIONAHA.125.24854 40905148 10.1161/HYPERTENSIONAHA.125.24854 PMC12529981

[14] National Institute for Health Research (NIHR). “Ethnicity data collection in NIHR research.” NIHR. 2021. Accessed August 2, 2025. https://www.nihr.ac.uk

[15] Boardman H Lamata P Lazdam M Verburg A Siepmann T Upton R Bilderbeck A Dore R Smedley C Kenworthy Y. ‘Variations in cardiovascular structure, function, and geometry in midlife associated with a history of hypertensive pregnancy’. Hypertension. 2020;75:1542–1550. doi: 10.1161/HYPERTENSIONAHA.119.14530 32306767 10.1161/HYPERTENSIONAHA.119.14530 PMC7682801

[16] Lazdam M de la Horra A Diesch J Kenworthy Y Davis E Lewandowski AJ Szmigielski C Shore A Mackillop L Kharbanda R. ‘Unique blood pressure characteristics in mother and offspring after early onset preeclampsia’. Hypertension. 2012;60:1338–1345. doi: 10.1161/HYPERTENSIONAHA.112.198366 23045462 10.1161/HYPERTENSIONAHA.112.198366

[17] National Institute for Health and Care Excellence (NICE). Hypertension in pregnancy: diagnosis and management (NG133). 2019. Accessed August 2, 2025. https://www.nice.org.uk/guidance/ng133 31498578

[18] Leung H Wang JJ Rochtchina E Tan AG Wong TY Hubbard LD Klein R Mitchell P. ‘Computer-assisted retinal vessel measurement in an older population: correlation between right and left eyes’. Clin Experiment Ophthalmol. 2003;31:326–330. doi: 10.1046/j.1442-9071.2003.00661.x 12880458 10.1046/j.1442-9071.2003.00661.x

[19] Streese L Lona G Wagner J Knaier R Burri A Nève G Infanger D Vilser W Schmidt-Trucksäss A Hanssen H. ‘Normative data and standard operating procedures for static and dynamic retinal vessel analysis as biomarker for cardiovascular risk’. Sci Rep. 2021;11:14136. doi: 10.1038/s41598-021-93617-7 34238996 10.1038/s41598-021-93617-7 PMC8266855

[20] Hubbard LD Brothers RJ King WN Clegg LX Klein R Cooper LS Sharrett AR Davis MD Cai J. ‘Methods for evaluation of retinal microvascular abnormalities associated with hypertension/sclerosis in the Atherosclerosis Risk in Communities Study’. Ophthalmology. 1999;106:2269–2280. doi: 10.1016/s0161-6420(99)90525-0 10599656 10.1016/s0161-6420(99)90525-0

[21] Lupton SJ Chiu CL Hodgson LAB Tooher J Lujic S Ogle R Wong TY Hennessy A Lind JM. ‘Temporal changes in retinal microvascular calibre and blood pressure during pregnancy’. Hypertension. 2013;61:880–885. doi: 10.1161/HYPERTENSIONAHA.111.00698 23399715 10.1161/HYPERTENSIONAHA.111.00698

[22] Ponto KA Werner DJ Wiedemer L Laubert-Reh D Schuster AK Nickels S Höhn R Schulz A Binder H Beutel M. ‘Retinal vessel metrics: normative data and their use in systemic hypertension: results from the Gutenberg Health Study’. J Hypertens. 2017;35:1635–1645. doi: 10.1097/HJH.0000000000001380 28505063 10.1097/HJH.0000000000001380

[23] Wong TY Klein R Klein BEK Meuer SM Hubbard LD. ‘Retinal vessel diameters and their associations with age and blood pressure’. Invest Ophthalmol Vis Sci. 2003;44:4644–4650. doi: 10.1167/iovs.03-0079 14578380 10.1167/iovs.03-0079

[24] Vatner SF Zhang J Vyzas C Mishra K Graham RM Vatner DE. ‘Vascular stiffness in aging and disease’. Front Physiol. 2021;12:762437. doi: 10.3389/fphys.2021.762437 34950048 10.3389/fphys.2021.762437 PMC8688960

[25] Humphrey JD. Mechanisms of vascular remodeling in hypertension. Am J Hypertens. 2021;34:432–441. doi: 10.1093/ajh/hpaa195 33245319 10.1093/ajh/hpaa195 PMC8140657

[26] Iorga RE Costin D Munteanu-Dănulescu RS Rezuș E Moraru AD. Non-invasive retinal vessel analysis as a predictor for cardiovascular disease. J Pers Med. 2024;14:501. doi: 10.3390/jpm14050501 38793083 10.3390/jpm14050501 PMC11122007

[27] Widmer RJ Lerman A. ‘Endothelial dysfunction and cardiovascular disease’. Glob Cardiol Sci Pract. 2014;2014:291–308. doi: 10.5339/gcsp.2014.43 25780786 10.5339/gcsp.2014.43 PMC4352682

[28] D’Amario D Migliaro S Borovac JA Restivo A Vergallo R Galli M Leone AM Montone RA Niccoli G Aspromonte N. ‘Microvascular dysfunction in heart failure with preserved ejection fraction’. Front Physiol. 2019;10:1347. doi: 10.3389/fphys.2019.01347 31749710 10.3389/fphys.2019.01347 PMC6848263

