# Supplementary material for: Retinal Vascular Caliber: 6 to 12 Months and 15 to 25 Years Following Hypertensive Pregnancy
Source: Hypertension. 2026 Jun 17;83(8):e27171. doi: 10.1161/HYPERTENSIONAHA.126.27171 (PMC13367563; doi:10.1161/HYPERTENSIONAHA.126.27171)
Supplement: Supplementary file 1 [file hyp-83-e27171-s001.docx]

**Supplementary Material for the Paper ‘Vascular Calibre: 6-12 Months and 15-25 Years Postpartum After Hypertensive Pregnancy’**

**INDEX OF SUPPLEMENTARY MATERIAL**

Supplementary methods
S1: Participant Flow Diagram.

***Correspondence to:** Professor Paul Leeson, Cardiovascular Clinical Research Facility, John Radcliffe Hospital, University of Oxford, Oxford. OX3 9DU, UK. Tel: +441865226829. E-mail : paul.leeson@cardiov.ox.ac.uk

**Supplementary methods**
**Inclusion criteria for the 6-12 month postpartum POP-HT hypertensive cohort**
• Is willing and able to give informed consent for participation in the trial.
• Is female, aged 18 years or above.
• Has a clinician confirmed diagnosis of either gestational hypertension or pre-eclampsia defined by the NICE guidelines (see below).
• Is requiring anti-hypertensive medication at the point of discharge from secondary care.
• Has clinically acceptable laboratory results and clinical course postpartum with no other adverse complicating factors requiring prolonged admission postpartum that would make their participation unfeasible as judged by the chief investigator. Examples would include stroke sequalae, ongoing disseminated intravascular coagulation etc.
• In the Investigator’s opinion, is able and willing to comply with all trial requirements including ownership of a ‘Smart-phone/Tablet’ and willing to use the smart-phone app if randomised to that arm.
• Has sufficient competence in English Language to follow the app instructions and partake in the study, as judged by the chief investigator.

**Diagnosis criteria for the 6-12 month postpartum POP-HT hypertensive cohort**
A diagnosis of gestational hypertension requires a blood pressure reading of more than 140 mmHg systolic or more than 90 mmHg diastolic on more than two occasions after 20 weeks of their current pregnancy.

A diagnosis of preeclampsia is defined as per the NICE guidelines and as follows: new onset of hypertension (more than 140 mmHg systolic or over 90 mmHg diastolic) after 20 weeks of pregnancy and the coexistence of one or more of the following new onset conditions:
• Proteinuria (spot urine protein/creatinine over 30 mg/mmol [0.3 mg/mg] or over 300 mg/day or at least one g/L [‘two +’] on dipstick testing) or other maternal organ dysfunction such as:

• Renal insufficiency (creatinine 90 umol/L or more, 1.02 mg/dL).

• Liver involvement (elevated transaminases [ALT or AST over 40 IU/L] with or without right upper quadrant or epigastric abdominal pain).

• Neurological complications such as eclampsia, altered mental status, blindness, stroke, clonus, severe headaches or persistent visual scotomata.

• Haematological complications such as thrombocytopenia (platelet count below 150,000 cells/µL), disseminated intravascular coagulation or haemolysis.

• Utero-placental dysfunction such as fetal growth restriction, abnormal umbilical artery Doppler waveform analysis, or stillbirth.

**Exclusion criteria for the 6-12 month postpartum POP-HT hypertensive cohort**
• Significant renal or hepatic impairment that would affect safe medication titration and adjustment as part of the trial, as deemed by the chief investigator.
• Scheduled elective surgery (excluding caesarean sections) or other procedures requiring general anaesthesia during the trial.
• A life expectancy of less than 6 months.
• Any other significant disease or disorder, which, in the opinion of the investigator, may either, put the participants at risk because of participation in the trial, or may influence the result of the trial, or the participant’s ability to participate in the trial.
• Participants who have participated in another research trial involving an investigational product in the last 12 weeks before their consent.
• An absolute contra-indication to magnetic resonance imaging (as per magnetic resonance imaging safety questionnaire) precludes the women from having a magnetic resonance imaging but they can still participate in the remainder of the study.
• Women with pre-existing hypertension will be excluded, as this is a separate pathology that would affect the efficacy of the study intervention and affect the primary and secondary outcomes of the study.

**Inclusion criteria for the 6-12 month postpartum CAREFOL-HT normotensive cohort**• Participant is willing and able to give informed consent for participation in the study.

• Aged more than 18 and less than or equal to 45 years.

• Normotensive, blood pressures < 140/90 mmHg throughout the entire antenatal period.

• Less than two moderate risk factors for hypertensive disease in pregnancy, according to the NICE guidelines for management of hypertension in pregnancy.

• SFlt/PIGF ratio of less than 35. 

**Exclusion criteria for the 6-12 month postpartum CAREFOL-HT normotensive cohort**• Diagnosis of a hypertensive disorder of pregnancy according to NICE guidelines.

• Use of beta blockers such as atenolol or equivalent.

•History of cardiac impairments including uncontrolled arrhythmia, unstable angina, decompensated congestive heart failure or valve disease.

• History of pre-existing chronic renal disease.

• Contraindication to magnetic resonance imaging e.g. pregnancy, pacemaker, ferromagnetic implant, shrapnel injury or severe claustrophobia.

• Any known trisomy, foetus with congenital heart defect, foetus at a high risk of heart disease, known infection of foetus or known severe anaemia.

**Inclusion criteria for the 15-25 year postpartum HELPFUL cohort**• Is willing and able to give informed consent for participation in the study.

• A female who had a pregnancy 10 to 25 years prior.

• Is 30 to 70 years of age.

• Is able (in the investigator’s opinion) and willing to comply with all study requirements.

• Has an adequate understanding of verbal and written English.

• Was either included as a case (hypertensive pregnancy) or control (normal pregnancy) in the Preeclampsia Vascular Study (PVS), or with the following features in obstetric records, as detailed below:

**Controls (normal pregnancy):**• Normal obstetric history (previous and index pregnancy).

• Diastolic blood pressure consistently below 90 mmHg.

• Systolic blood pressure consistently less than 140 mmHg.

• No more than a trace of proteinuria.

**Cases only (Hypertensive Pregnancy):**• New onset hypertension after 20 weeks of gestation in index pregnancy.

• Diastolic blood pressure of more than 90 mmHg on two separate occasions within a 24-hour period.

**Pre-eclampsia or Hypertension with Superimposed Pre-eclampsia**• Raised blood pressure (as above) and new onset proteinuria of:

• 300 mg/24 h or more of protein in a 24-hour urine collection.

• More than 30 mg of protein/mmol of creatinine in a single urine sample.

• Or at least two+ protein at least twice on consecutive dipstick testing.

**Exclusion criteria for the 15-25 year postpartum HELPFUL cohort**
The participant may not enter the study if ANY of the following apply:
• Pregnant or lactating when they are due to attend for the study visit.
• Planning to donate blood within two weeks prior to the study visit.
•Has evidence of congenital heart disease or significant chronic disease relevant to cardiovascular or metabolic status.
•Has any significant disease or disorder which, in the opinion of the investigator, might influence the participant’s ability to participate in the study

For exclusion of MRI component only:
• Is unsuitable for magnetic resonance imaging based on participant screening. The participant may still be included in other parts of the study.

**Anthropometry measurements**
Height and weight were measured, after removal of shoes and heaving clothing, to the nearest centimetre and 0.1 kg, respectively. Height was measured on a free-standing stadiometer and weight was measured on scales. Body mass index (BMI) was calculated using the formula: BMI (kg/m^2^) = weight (kg) / height (m)^2^. Hip circumference was measured at the widest part of the gluteal region, whilst waist circumference was measured just above the belly button. Waist to hip ratio was then calculated. Left arm circumference was measured at the widest part of the upper arm while relaxed.

**
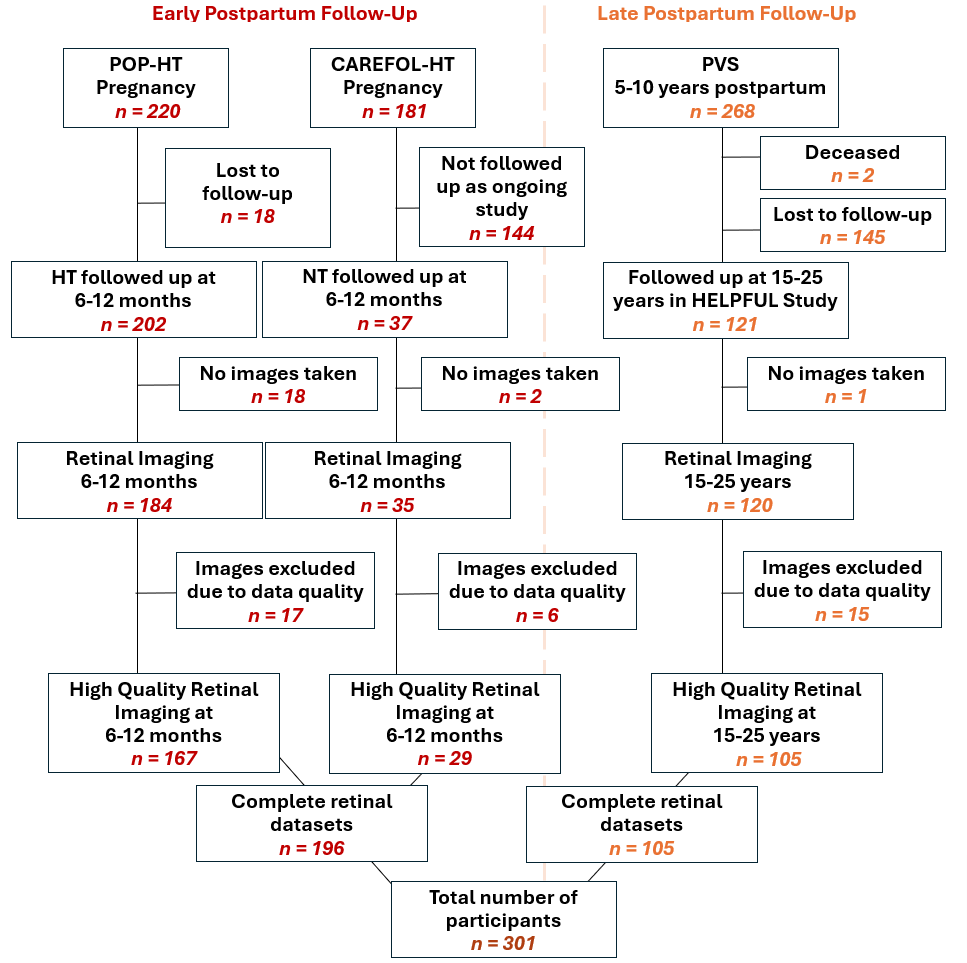
**

**S1: *Participant Flow Diagram.*** This diagram illustrates the participant selection process and the number of individuals included at each stage of the study. The study involved recruitment from three original cohorts: POP-HT (n = 220), CAREFOL-HT (n = 181), and PVS (n = 268). The total number of participants with retinal imaging data included in the final analysis was 301.
